# Association between Mastication, the Hippocampus, and the HPA Axis: A Comprehensive Review

**DOI:** 10.3390/ijms18081687

**Published:** 2017-08-03

**Authors:** Kagaku Azuma, Qian Zhou, Masami Niwa, Kin-ya Kubo

**Affiliations:** 1 Department of Anatomy, School of Medicine, University of Occupational and Environmental Health, 1-1 Iseigaoka, Yahatanishi-ku, Kitakyushu, Fukuoka 807-8555, Japan; zhou@med.uoeh-u.ac.jp; 2 Department of Radiology, JA Gifu Welfare Federation Ibi Welfare Hospital, 2547-4, Miwa Ibigawa-cho, Ibi-gun, Gifu 501-0696, Japan; m7niwa@poem.ocn.ne.jp; 3 Department of Food Science and Nutrition, Faculty of Human Life and Environmental Science, Nagoya Women’s University, 3-40 Shioji-cho, Mizuho-ku, Nagoya, Aichi 467-8610, Japan; kubo@nagoya-wu.ac.jp

**Keywords:** chewing, masticatory dysfunction, hippocampus, HPA axis

## Abstract

Mastication is mainly involved in food intake and nutrient digestion with the aid of teeth. Mastication is also important for preserving and promoting general health, including hippocampus-dependent cognition. Both animal and human studies indicate that mastication influences hippocampal functions through the end product of the hypothalamic-pituitary-adrenal (HPA) axis, glucocorticoid (GC). Epidemiologic studies suggest that masticatory dysfunction in aged individuals, such as that resulting from tooth loss and periodontitis, acting as a source of chronic stress, activates the HPA axis, leading to increases in circulating GCs and eventually inducing various physical and psychological diseases, such as cognitive impairment, cardiovascular disorders, and osteoporosis. Recent studies demonstrated that masticatory stimulation or chewing during stressful conditions suppresses the hyperactivity of the HPA axis via GCs and GC receptors within the hippocampus, and ameliorates chronic stress-induced hippocampus-dependent cognitive deficits. Here, we provide a comprehensive overview of current research regarding the association between mastication, the hippocampus, and HPA axis activity. We also discuss several potential molecular mechanisms involved in the interactions between mastication, hippocampal function, and HPA axis activity.

## 1. Introduction

For survival, all living organisms must maintain a complex dynamic homeostasis that is constantly challenged by various external and internal disturbances or stressors. The organismal response to various stressors represents an integrated reaction directed to maintain homeostasis [1,2]. The main neuroendocrine response to stress is via activation of the hypothalamic-pituitary-adrenal (HPA) axis [2,3,4,5]. The hypothalamic paraventricular nucleus (PVN), a central component of the HPA axis, contains neurons that secrete corticotropin-releasing hormone (CRH) and arginine vasopressin (AVP) [6,7]. The hypothalamic PVN receives many afferent inputs from various regions of the brain, including the hippocampus, amygdala, and prefrontal cortex, and integrates information obtained from the peripheral sensory organs that is then assembled in these brain regions [8,9]. CRH and AVP are released from the hypothalamic median eminence into the pituitary portal system to promote the secretion of adrenocorticotrophic hormone (ACTH). Circulating ACTH then stimulates the synthesis and secretion of glucocorticoid (GC) from the adrenal cortex [2,8]. Adrenal GC secretion shows strong circadian rhythms with peak levels during the activity period. By altering the expression of circadian genes, GCs are demonstrated to affect the circadian rhythm system in influencing daily gene expression upon exposure to stressors [10]. GCs include corticosterone in rodents, and cortisol, the predominant GC in humans. GCs act upon their receptors, which are present in almost all tissues and organs of the body, initiating metabolic, physiological and behavioral actions. GCs regulate a plethora of physiological processes, including intermediary metabolism, immune function, skeletal growth, cardiovascular function, reproduction, and cognition [11,12]. GCs promote hepatic gluconeogenesis and increase glycogen storage in liver. Excess GC exposure causes hyperglycemia and insulin resistance [13]. GCs also modulate the intestinal epithelial barrier function, which is pivotal to the pathogenesis of the inflammatory bowel diseases [14]. Adequate activation of the HPA axis is critically important for stress adaptation. Repeated or prolonged HPA axis hyperactivity, however, is linked with numerous physiological and psychological disorders [6].

Mastication is the process by which food is crushed and ground by the teeth. It is mainly involved in food intake and digestion. Mastication is highly important, not only for food intake, but also for promoting and preserving general health [15,16,17]. Masticatory dysfunction resulting from tooth loss; periodontitis; and an inadequate vertical dimension of crowns, bridges, or dentures has physical, psychological, and social effects on the quality of life. Numerous human and animal studies indicate that masticatory dysfunction, acting as source of chronic stress, activates the HPA axis, leading to increases in circulating GC levels and precipitating various illnesses, such as hippocampus-dependent cognitive deficits, hypertension, cardiovascular disorders, and osteoporosis [18,19,20,21]. Recent studies indicate that masticatory stimulation or chewing during stressful conditions can ameliorate chronic stress-induced physical and psychological disorders by suppressing HPA axis hyperactivity [15,16,17,22,23].

Masticatory stimulation has important functions related to physical, mental, and social health [15,16,24,25]. Masticatory ability affects the nutritional state, overall health, and daily living activity, especially in the elderly population [26]. Many elderly adults have dental problems due to tooth loss, which is a risk factor for senile dementia [27,28,29,30,31,32,33]. In animals, masticatory dysfunction induced by removing teeth leads to hippocampus-dependent spatial memory and learning deficits [15,16,34,35,36,37,38]. Therefore, chewing appears to have an important role in maintaining hippocampus-dependent cognitive function [15,16,17,27]. Chewing is also an effective stress-coping behavior. Chewing or biting on objects is considered to help relieve emotional tension or stress. Rats or mice provided the opportunity to chew on wooden sticks under stressful conditions exhibit attenuated HPA axis activity and reduced circulating GC levels, which helps to prevent stress-induced cognitive deficits, gastric ulcers, and osteoporosis [15,16,20,21,39]. Here, we provide a comprehensive overview of the current understanding of the relationship between mastication, the hippocampus, and HPA axis activity. The organization of the HPA axis is highly conserved throughout the mammalian phylogeny and most fundamental aspects of the HPA axis are similar across the phylogenic trajectory from rodents to humans. Therefore, this review focuses largely on rodent studies.

## 2. Masticatory Dysfunction Activates the Hypothalamic-Pituitary-Adrenal (HPA) Axis

Previous studies using animal models elucidated a possible link between masticatory dysfunction and HPA axis activity [15,16,17]. Masticatory dysfunction activates the HPA axis, affecting physical, psychological, and social health, contributing to senile dementia, Alzheimer’s disease, and a declining quality of life in the elderly. Tooth loss and inappropriate vertical dimensions of crowns, bridges, or dentures can induce masticatory dysfunction in humans. Masticatory dysfunction may cause bruxism, headache, or pain in the masticatory muscles and temporomandibular joint, and general malaise [26,30,33,40]. Masticatory dysfunction sustainably activates the HPA axis and increases circulating GC levels. The continuous increase in circulating GC levels induced by masticatory dysfunction disrupts the negative feedback system of the HPA axis, further enhancing the secretion of GCs.

### 2.1. Occlusal Disharmony and the HPA Axis

Animal studies suggest an association between occlusal disharmony, stress, and HPA axis activation. Experimental occlusal disharmony in animals induced by applying adhesive to the molar teeth, attaching acrylic caps at the incisors, or inserting occlusal splints in the maxilla, quickly increases the circulating and urine corticosterone levels as acute stress responses that persist for weeks. In monkeys, urinary cortisol excretion rates are elevated by the insertion of occlusal splints [41]. In rats, occlusal disharmony produced by placing acrylic caps on both lower incisors increases circulating corticosterone levels [42]. In mice, occlusal disharmony is induced by the bite-raising procedure performed under anesthesia, in which the vertical dimension of the bite is raised by approximately 0.1 mm by applying ultraviolet-ray polymerization resin to the upper molars after treatment with a single bond dental adhesive system [37,38,43,44,45,46,47]. Eight days after the procedure, the HPA axis is activated and the circulating corticosterone levels are increased, with significant increases detected only in old bite-raised mice [15,17,35,37]. Masticatory dysfunction may not affect HPA axis activity in young mice, as the brain receives rich sensory inputs continuously through the peripheral sensory organs and vigorous locomotor activity to maintain its function. In old age, however, senesced peripheral organs and decreased locomotor activity do not provide sufficient sensory input to maintain the normal feedback regulation of the HPA axis, leading to HPA axis hyperactivity [5,48,49]. These findings suggest that masticatory dysfunction induced by bite-raised condition or occlusal disharmony results in HPA hyperactivity, and increased circulating GC levels. The sustained increase in circulating GC levels by masticatory dysfunction impairs negative feedback regulation of the HPA axis.

### 2.2. Tooth Extraction and the HPA Axis

Tooth loss due to dental caries and periodontitis are common in the elderly population. Permanent tooth loss decreases the somatosensory stimuli from the oral cavity, inducing sustained increases in circulating GCs. Several studies demonstrated that loss of molar teeth for a long period of time in rodents induces chronic psychological stress [36,50,51,52,53,54,55,56,57]. We used dental tweezers to bilaterally remove the maxillary molars in senescence accelerated mouse prone 8 (SAMP8) mice at the age of one month [34,35,36,50,51,52,53,57,58,59]. SAMP8 mice undergo normal maturation up to the age of six months, and then exhibit accelerated aging with a median life span of 12 months compared with two or three years for other strains. SAMP8 mice are a proposed experimental murine model for human senile dementia [34,35,36,50,51,52,53,57,58,59]. Circulating corticosterone levels were measured in young, adult, and old mice after tooth extraction. The results demonstrated that circulating corticosterone levels increase with age. The circulating corticosterone levels were not significantly different between the young control and toothless mice, whereas circulating corticosterone levels were significantly higher in adult and old toothless mice than in the age-matched control mice [15,16,55]. As chronic stress causes a significant increase in the circulating corticosterone levels, we consider that tooth loss may act as chronic stressor in adult and aged SAMP8 mice [20,35,57,59].

### 2.3. Masticatory Dysfunction and Hippocampal Function

Many reports link stress and reduced hippocampal neurogenesis. Acute and chronic stress exposure impairs hippocampal neurogenesis, reduces progenitor cell proliferation, and suppresses neuronal differentiation and cell survival in the hippocampal dentate gyrus [15,16,45,47,58]. In addition to these direct effects of acute and chronic stress on hippocampal progenitor cells in affected animals, prenatal stress and exposure to excess GCs in utero alter the brain development and maturation of littermates, which can result in adverse consequences in later adult life, reducing the lifespan of neurogenesis in the hippocampal dentate gyrus, and leading to hippocampus-dependent cognitive deficits [21,22,23]. These findings indicate that stress not only affects adult hippocampal neurogenesis, but also has a transgenerational effect through transmitted epigenetic mechanisms, leading to lifelong effects in littermates when the pregnant mother is exposed to stressful conditions [60].

Animal studies demonstrated that the circulating corticosterone levels are significantly higher in toothless and bite-raised mice than in control mice. Pretreatment with metyrapone, a corticosterone synthesis inhibitor that suppresses the stress-induced increase in circulating corticosterone levels, prevents the toothless condition-induced increase in circulating corticosterone [51]. Chronic stress induces adrenal gland enlargement and increases circulating corticosterone levels [20,61,62]. Adrenalectomy abolishes stress-induced bone loss, implicating corticosterone in this effect [62]. GC-induced osteoporosis is the most common form of secondary osteoporosis. GCs have detrimental effects on bone by suppressing osteoblastic bone formation and activating osteoclastic bone resorption via glucocorticoid receptors (GRs) in bone cells.

Sustained masticatory dysfunction induced by the toothless condition at an early age accelerates the aging process of hippocampus-dependent cognitive function at an advanced age. Circulating corticosterone levels are significantly higher in mature and old toothless mice. As chronic stress causes a significant increase in circulating corticosterone, we consider that tooth loss early in life may act as chronic stressor in adult and aged mice. Emerging evidence from neuroimaging studies suggests that masticatory dysfunction causes hippocampus-dependent cognitive impairment [63,64]. Converging evidence from animal studies indicates that masticatory dysfunction induced by occlusal disharmony or tooth extraction impairs spatial memory and learning ability in rats and mice [15,16,17,37,38,39,40,48,49,50,51,53,54,55]. These animals are able to bite, but masticatory function is markedly impaired, thus causing degenerative alterations of the periodontal sensory receptors [48]. Morphologic studies revealed that hippocampal neurons, dendritic spines, and post-synaptic densities are significantly decreased in toothless or bite-raised rodents [23,34,35,37,38,43,45,47]. Immunohistochemical studies revealed that prolonged masticatory dysfunction suppresses neurogenesis in the hippocampal dentate gyrus. Toothless or bite-raised animals exhibit significantly reduced neuronal proliferation, newborn cell survival, and cell differentiation in the hippocampal dentate gyrus in an age-dependent manner [22,45,47,58,59]. Experiments using the Morris water maze test showed that toothless and bite-raised animals have significantly longer escape latencies and impaired spatial memory and learning ability [22,23,43,45,47,58,59]. These findings suggest that masticatory dysfunction impairs cell proliferation in the hippocampal dentate gyrus, causing hippocampus-dependent cognitive deficits.

### 2.4. Molecular Links between Masticatory Dysfunction and HPA Axis Hyperactivity

The hippocampus and other limbic system structures comprise the neural circuitry that forms an adaptive response to stress through communicating with other brain structures, such as the hypothalamus and prefrontal cortex [65]. The neurogenesis of the hippocampal dentate gyrus is also critical for hippocampus-mediated negative modulation of the HPA axis [66]. Previous studies demonstrated that bite-raised animals have increased circulating corticosterone levels associated with spatial learning deficits [37,43,44,45,46,47]. CRH and AVP are secreted by parvocellular neurons in the hypothalamic PVN, and regulate pituitary ACTH secretion. AVP alone only weakly induces ACTH secretion, but it has synergistic actions with CRH and helps to sustain the pituitary response during chronic stress. The parvocellular PVN contains two populations of CRH neurons, one containing CRH alone and the other colocalized with AVP [7,9]. Secretion of CRH and AVP is stimulated by acute stress. In repeated or chronic stress conditions, neurons in which CRH and AVP colocalize preferentially secrete AVP. An in situ hybridization study showed that the bite-raising procedure in mice induces a rapid increase in CRH mRNA expression and a slower increase in AVP mRNA expression in the hypothalamic parvocellular PVN. Exposure to a novel stress in the bite-raised condition further reinforces the CRH stress response. Consequently, masticatory dysfunction induced by the bite-raised condition may be a risk factor for hypersensitivity to novel stress [46].

**Figure 1:**
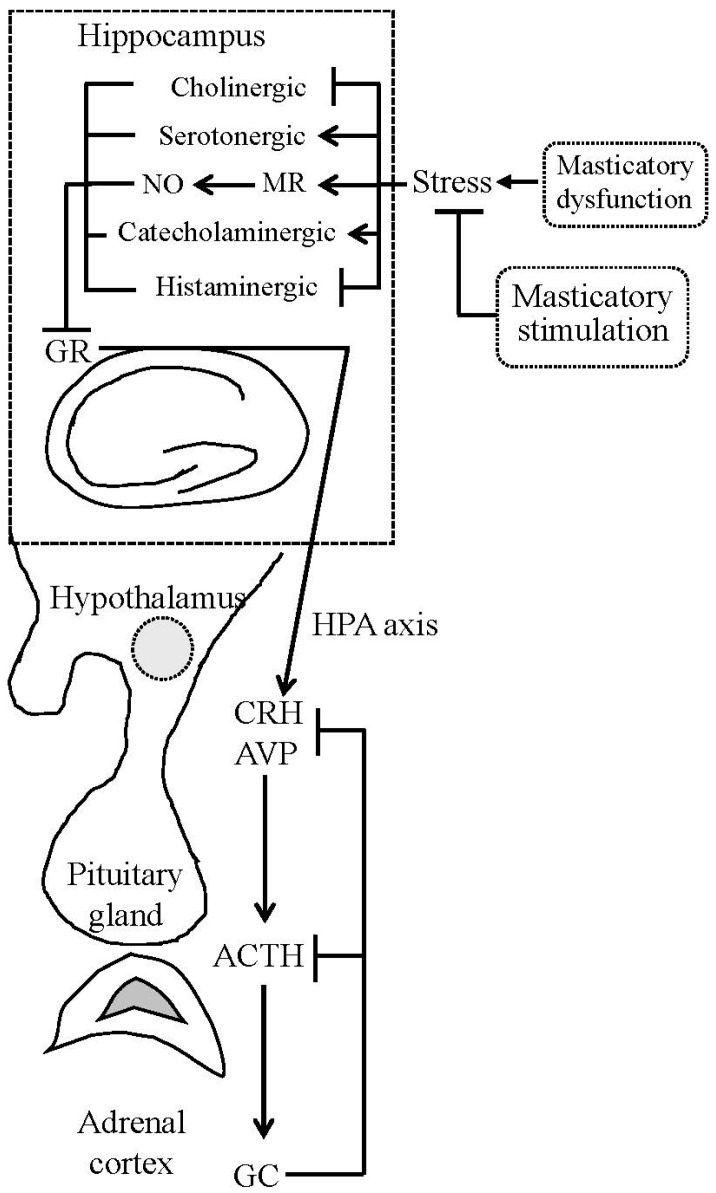
Simplified diagram showing the relationship between mastication, the hippocampus, and hypothalamic-pituitary-adrenal (HPA) axis activity. Masticatory dysfunction, acting as a stressor, suppresses hippocampal choline and histamine levels, and elevates catecholamine, serotonin, nitric oxide (NO) levels, and mineralocorticoid receptor (MR) expression, and then reduces the hippocampal glucocorticoid receptor (GR) expression, leading to hypersecretion of hypothalamic corticotropin-releasing hormone (CRH) and arginine vasopressin (AVP), and HPA axis hyperactivity. Masticatory stimulation suppresses the hyperactivity of the HPA axis and thus ameliorates stress-induced disorders. ACTH, adrenocorticotrophic hormone; GC, glucocorticoid.

Sustained stress, such as tooth loss and occlusal disharmony via GC hypersecretion, induces hippocampal damage. The stress response is also modulated by a number of neurotransmitters and neuropeptides that interact with the HPA axis (Figure 1). The molecular interactive link between GCs and the cholinergic system contributes to stress-induced hippocampus-dependent cognitive deficits with aging [17,67]. Basal forebrain cholinergic neurons provide inputs to the hippocampus. The hippocampus along with cholinergic innervation from the basal forebrain is involved in regulating the stress response of the HPA axis. Stress suppresses the septohippocampal cholinergic pathway, which causes acetylcholine-mediated responses by stimulating HPA axis activity [68]. In toothless or bite-raised aged mice, the number of choline acetyltransferase-immunopositive neurons in the medial septal nucleus is decreased. Hippocampal acetylcholine release, acetyltransferase, and choline acetyltransferase activity are significantly decreased in old animals with masticatory dysfunction [35,44]. Masticatory dysfunction in young animals, however, has few effects, indicating an age-dependent decline in the hippocampal cholinergic system. These findings indicate a possible intricate interaction between GCs, the HPA axis, and the septohippocampal cholinergic pathway.

Nitric oxide (NO) is produced by nitric oxide synthase (NOS), including endothelial NOS, inducible NOS, and neuronal NOS (nNOS), which is the main form expressed in the brain, localized to the sites of neuronal proliferation and migration in the hippocampal dentate gyrus and forebrain subventricular zone. The nNOS-derived NO has multiple functions, including neurogenesis, synaptogenesis, and neural plasticity [69]. It is implicated in the regulation of various behavioral, cognitive, and emotional processes. Animals exposed to prolonged or chronic stress have elevated corticosterone levels in both the hippocampus and hypothalamus [70]. The balance between the GR and mineralocorticoid receptor (MR) expression levels is considered an important factor in resilience to stress. GRs and MRs are highly colocalized in the hippocampus of almost all species. MRs bind GCs with high affinity and thus are already occupied at low GC levels. GRs, in contrast, have 10-fold lower affinity and are therefore only occupied under conditions of high GC levels [71]. GRs are widely distributed throughout the brain, while MRs localize mainly in limbic brain structures, like the hippocampus, lateral septum, amygdala, hypothalamus, and medial prefrontal cortex [72]. Hippocampal nNOS upregulation by GCs is dependent on MR activation. There are no obvious differences in the GR expression levels between the hippocampus and hypothalamus. MR expression in the hippocampus, however, is markedly higher than that in the hypothalamus. MR selectivity of the hippocampal nNOS expression may result, however, as MRs bind GCs with higher affinity than GRs.

Animals exposed to chronic stress initially exhibit increased MR expression [73] and nNOS in the hippocampus, impaired hippocampal neurogenesis, and hippocampus-dependent behavioral changes. These alterations are prevented and reversed in mutant mice lacking the nNOS gene and in mice receiving the nNOS inhibitor [74]. Recent studies showed that exposure to chronic stress upregulates hippocampal nNOS expression by activating MR expression, leading to downregulation of the hippocampal GRs via cyclic guanosine monophosphate (cGMP)-dependent and cGMP-independent mechanisms [70]. Chronic stress-induced elevation of hypothalamic CRH secretion by hippocampal corticosterone microinjection is not due to hypothalamic nNOS alteration [75]. Consequently, it is conceivable that alterations of the hippocampal GR expression level contribute to hyperactivity of the HPA axis under chronic stress. The increased hippocampal corticosterone level activates the MR-nNOS-NO signaling pathway, causing disruption of GR expression, and finally inducing the hyperactivity of the HPA axis [70,76]. The elevated CRH secretion will persistently stimulate HPA axis activity, eventually leading to increased circulating GC levels [77]. Repeated or chronic exposure to GCs has detrimental effects on the brain, especially the hippocampus, which is essential for HPA axis restraint, as well as memory and cognitive consolidation.

The hippocampus receives serotonin (5-HT) neuronal projections from the raphe nucleus, which is the major origin of the central 5-HT system. Expression and function of the hippocampal GRs and MRs are regulated by central 5-HT receptors [78,79]. Stressful stimuli increase hippocampal 5-HT release and turnover. Some of the changes in MR and GR expression may be mediated, at least in part, by an increase in the hippocampal 5-HT levels [78]. Accumulated evidence indicates that depressed patients exhibit 5-HT1 and 5-HT2 receptor function abnormalities, which may then act to dysregulate HPA axis function. As the rate-limiting enzyme for the synthesis of central 5-HT, tryptophan hydroxylase 2 (TPH2) is a key player in the modulation of 5-HT neurotransmission and is thus a potential target for therapeutic treatment of psychiatric disorders [78,79,80]. GCs affect TPH2 expression and 5-HT neurotransmission in mice and rats. Expression of the *TPH2* gene is sensitive to various stresses, because of the stress-induced increases in GC levels [81]. Conflicting findings are reported regarding the effect of stress-exposure on TPH2 expression in the animal brain. Exposure of mice to chronic stress elevates *TPH2* mRNA expression in the dorsal raphe and median raphe nuclei [79]. In contrast, repeated treatment with GCs decreases raphe *TPH2* mRNA expression in mice [82]. These findings emphasize the relevance of *TPH2* gene expression to the stress response. TPH2 plays an important role in the regulation of 5-HT neurotransmission and is closely related to the stress response.

## 3. Masticatory Stimulation Preserves Hippocampal Function

Several human studies indicate that masticatory stimulation or chewing modifies the effects of stress and is effective for preserving hippocampus-dependent cognitive function, which deteriorates with aging. Population-based studies demonstrated an association between masticatory ability or oral health and cognitive functions [27,28,29,30,31,32,33]. Animal studies indicate that actively chewing on a wooden stick during immobilization stress ameliorates the stress-induced impairment of synaptic plasticity and prevents stress-induced noradrenaline release in the amygdala [15,16]. Previous studies suggested that the prefrontal cortex dominantly regulates the stress response system, including the HPA axis. Gum-chewing influences the brain physiological and psychological responses, and evokes activation of the prefrontal cortex. Activation of the prefrontal cortex might also influence HPA axis activities [83]. Chewing increases cerebral blood flow and might thus decrease the risk of cognitive impairments [84]. Chewing itself may positively affect cerebral blood flow, alleviate stress, and therefore enhance cognitive function. A functional magnetic resonance imaging study indicated that chewing influences cerebellar function, which is involved in chewing rhythmicity and motor control motivated by proprioceptive inputs. The enhanced cerebellar function during chewing may also improve hippocampus-dependent cognition [63,64].

Chewing is an effective stress-coping behavior. When exposed to inescapable stressors, animals assume coping behaviors, such as chewing, that attenuate some elements of the stress response. Animal studies have demonstrated that chewing or biting wooden sticks during immobilization or restraint stress decreases the stress-induced circulating corticosterone levels and attenuates HPA axis hyperactivity, which helps to prevent the stress-induced formation of gastric ulcers, deficits in spatial learning ability, and bone loss [15,16,17,20,21,22,23]. As the hippocampus is a target region for stress hormones, regulating its feedback control system, attenuated hippocampal function may further lead to unregulated corticosterone secretion [15,16].

Animal studies have also demonstrated that a soft diet suppresses hippocampal neurogenesis, while subsequent hard diet feeding improves neurogenesis in mice [85]. Hippocampus-dependent spatial learning deficits induced by the toothless condition are considerably improved by restoring lost teeth with artificial crowns, even in aged mice [50]. Chewing under immobilization or restraint stress reverses the stress-induced impairments in neurogenesis in the hippocampal dentate gyrus and attenuates the effect of stress on cognitive function [15,16,22,23]. Chewing under various stress conditions significantly suppresses the stress-induced elevation of CRH expression in the hypothalamic PVN. Chewing activates the hippocampal GR-immunopositive neurons suppressed by stress, thus improving disrupted HPA axis activity [86,87,88].

A possible molecular mechanism for chewing-induced alterations in hippocampus-related changes is the brain histaminergic reaction. Chewing under stress stimulates histaminergic neurons in the tuberomammillary nucleus (TMN). Axons of histaminergic neurons in the TMN project widely throughout the brain, including the hippocampus [89]. Activation of the mesencephalic trigeminal nucleus by chewing stimulates histaminergic neurons in the TMN of the posterior hypothalamus, thereby increasing histamine levels within the brain [90,91]. Electrical stimulation of the TMN enhances hippocampal histamine levels [92]. Chewing-induced increases in hippocampal histamine levels might rescue stress-attenuated hippocampal synaptic plasticity and hippocampus-dependent cognitive processes via facilitating *N*-methyl-d-aspartate receptor activity [17,93]. Blockade of histamine H1 receptors antagonizes the effect of chewing on synaptic plasticity [94], suggesting that chewing induces increases in hippocampal histamine levels, and restores the stress-attenuated hippocampal memory processes mediated by histamine H1 receptors.

Masticatory stimulation during stress prevents stress-induced noradrenaline release within the amygdala [94,95]. Using Fos-immunoreactivity as a measure of neuronal activation, Stalnaker et al. [96] found that chewing during stress suppresses Fos expression in the amygdala and increases Fos expression in the prefrontal cortex, where they previously demonstrated a chewing-attenuated dopaminergic response to stress [97]. As the prefrontal cortex plays a pivotal role in cognition and affective processes, and as dopaminergic neurotransmission in this region is regulated by the amygdala, these findings suggest that chewing suppresses neuronal transmission in the amygdala to further attenuate stress-related dopamine release in the prefrontal cortex. Chewing during stress leads to rapid recovery of nigrostriatal dopaminergic activity to the resting level [98]. These findings provide evidence that masticatory stimulation modulates catecholaminergic neurotransmission in the brain to regulate the perception of stress, possibly altering affective states.

## 4. Masticatory Stimulation Attenuates HPA Axis Activation

Like occlusal disharmony and tooth loss, immobilization and restraint act as stressors, activating the HPA axis. Restraint stress induced by placing mice in a ventilated plastic restraint tube in which they can move back and forth, but not turn around, significantly increases circulating corticosterone levels. Restrained mice allowed to chew on a wooden stick as masticatory stimulation simultaneously during the experimental period exhibit an attenuated increase in circulating corticosterone levels [20,22,23]. Increases in circulating ACTH and corticosterone levels induced by immobilization stress are suppressed by chewing a wooden stick [99,100]. Masticatory stimulation or chewing during stressful conditions suppresses the hyperactivity of the HPA axis (Figure 1). In rodents, chewing or biting on wooden sticks under various stressful conditions, such as immobilization, restraint, cold exposure, and tail pinch, attenuates the secretion of ACTH [17,99,100,101] and increases in circulating corticosterone levels.

Recent studies reported that rodents given wooden sticks to chew under stressful conditions have attenuated stress-induced memory impairment and hippocampal GR expression [63,64,85,86]. Chronic stress causes the downregulation of GR expression and inhibition of the negative feedback system from the hippocampus to the HPA axis [102]. Masticatory stimulation under stressful conditions significantly suppresses the stress-induced increase of CRH expression in the hypothalamic PVN [103]. Masticatory stimulation could attenuate the hyperactivity of the HPA axis, improve the ability to cope with stress, and alleviate chronic stress-induced hippocampus-dependent cognitive deficits. Aging is associated with an increase in the variability and disturbances in the HPA axis. Hyperactivity of the HPA axis is detected at all levels of the HPA organization with aging [104]. Gum chewing reportedly improves the performance of memory recall in elderly subjects, but not in young subjects [17,63,64]. Masticatory stimulation is more effective in aged animals compared with young animals, as reported previously [15,16]. Effects of masticatory stimulation on HPA axis activity via the hippocampus involve multiple pathways, including the peripheral sensory nervous inputs, stress hormones and their receptors, a number of neurotransmitters and neuropeptides, and the autonomic nervous system (Figure 1). Elucidation of the precise mechanism is anticipated in the near future.

## 5. Conclusions

Mastication plays an important role in regulating HPA axis activity and conserving hippocampus-dependent cognitive function. Masticatory dysfunction leads to hippocampal impairment through inducing hyperactivity of the HPA axis and various neural circuits, leading to hippocampus-dependent spatial learning and memory deficits. Masticatory stimulation or chewing during stress conditions could improve stress-induced hippocampal neurogenesis, synaptic plasticity, and cognitive function by attenuating stress hormones and their receptors, HPA axis activity, and several signaling pathways. Therefore, masticatory stimulation might be an effective method for modulating the normal feedback mechanism of the HPA axis and preventing various stress-induced disorders, especially in older people.

The association between mastication, stress, hippocampus, and the HPA axis activity is complicated, involving catecholaminergic, cholinergic, histaminergic, and 5-HT neurotransmitter systems; NO signaling; and alterations of MR and GR expression. Further studies are needed to clarify the detailed mechanisms underlying the HPA axis activity mediated by mastication.
